# Application and challenges of ChatGPT in interventional surgery

**DOI:** 10.1097/JS9.0000000000000704

**Published:** 2023-09-13

**Authors:** Zhaorong Wu, Linke Bian, Haigang Geng, Zhigang Zheng, Hongye Wang, Bo Zhai

**Affiliations:** a Department of Interventional Oncology; b Department of Gastrointestinal Surgery, Renji Hospital, Shanghai Jiao Tong University School of Medicine; c Department of Liver Surgery and Liver Transplantation, School of Medicine, Renji Hospital, Shanghai Jiao Tong University, Shanghai, People’s Republic of China

With the rapid developments of artificial intelligence (AI), powerful AI-related technologies have gradually permeated into many areas of life, bringing transformative changes and innovations to diverse domains. ChatGPT is an AI language model developed by OpenAI. Trained on an extensive dataset, it exhibits formidable natural language processing (NLP) capabilities. Employing cutting-edge deep learning techniques, ChatGPT engages in conversations, responds to queries, and generates human-like text. Its applications span various domains, including education, healthcare, research, and customer service, with relevance extending to the field of interventional surgery and beyond^1^. Exercising careful consideration is imperative, given that ChatGPT’s responses may not consistently encompass exhaustive accuracy, particularly in the context of interventional surgery. While its purpose is to assist and facilitate, users should verify critical information from credible sources when necessary. In recent months, several studies have showcased the significant potential of ChatGPT/GPT-4.0 in the field of medicine, such as providing medical information and research retrieval, assisting with clinical decision-making, educating and engaging patients, analyzing medical records with NLP, identifying drug interactions and safety issues, offering medical education and training, and supporting mental health inquiries^2^. To the best of our knowledge, no study has yet evaluated the potential impact of ChatGPT, particularly GPT-4, in the domain of interventional surgery. Exploring the possibilities of how ChatGPT could offer assistance in the field of interventional surgery has become an essential topic.

In the field of interventional surgery, our department specializes in procedures such as radiofrequency ablation (RFA), transarterial chemoembolization (TACE), microwave ablation (MWA), cryoablation, particle implantation, and irreversible electroporation for liver tumor treatment. Therefore, from this standpoint, we offer recommendations for integrating ChatGPT into these interventions. Preoperatively, ChatGPT can analyze patient data encompassing medical records, imaging, and tumor characteristics. This analytical insight assists doctors in devising and individualizing procedures like RFA, TACE, or MWA to align precisely with each patient’s distinct condition. Furthermore, ChatGPT potentially provides the capability of conducting virtual simulations and generating 3D visualizations of the designated areas. This attribute assists doctors in comprehending the intricacies of the procedure, fine-tuning their approach, and enhancing strategy refinement in anticipation of the actual surgical intervention. During surgeries, ChatGPT emerges as a real-time guide, lending its support to surgeons as they navigate intricate anatomy, ascertain optimal needle placement, and navigate dynamic conditions. Moreover, it serves as a tool for risk assessment, adeptly evaluating potential hazards and complexities entailed in RFA, TACE, or MWA. This resource empowers surgeons to make informed, prudent choices that mitigate risks and ensure safer treatment avenues. In the postoperative phase, ChatGPT continues to demonstrate its utility. It offers valuable assistance by monitoring patients’ progress after surgery, assessing the efficacy of treatments, and providing recommendations for suitable follow-up procedures or interventions. This continuous involvement highlights ChatGPT’s function as a versatile resource that supports the entire process, from preoperative to postoperative stages. In the realm of medical education, ChatGPT emerges as a multifaceted learning tool, granting access to cutting-edge research, guidelines, and case studies encompassing RFA, TACE, and MWA^3^. This provision empowers the continuum of professional advancement. Additionally, when collaboration with specialized experts is essential, ChatGPT facilitates seamless remote consultations and fosters the exchange of knowledge among interventional surgeons. Beyond professional collaboration, ChatGPT extends its influence to patient education. It offers simplified explanations of complex procedures, enhancing patient comprehension and alleviating apprehensions. This multifunctional engagement underscores ChatGPT’s capacity to drive informed decision-making, elevate medical practice, and bridge the gap between professionals and patients.

Leveraging the synergy of deep learning and big data technology, ChatGPT holds the potential to enhance the efficiency and accuracy of medical image analysis, including X-ray and computed tomography (CT) scans. Equipped with well-trained deep learning models, ChatGPT can autonomously recognize and segment diverse structures within images, such as organs, tumors, and blood vessels. Additionally, extracting and calculating various parameters from medical images, such as tumor volume and vascular constriction, could facilitate disease assessment, predict disease progression, and offer insights into patient prognosis^4^. Amidst surgical procedures, it possesses the capacity to provide real-time navigation information, encompassing anatomical structures, procedural guidance, and more. This capability aids surgeons in making precise decisions throughout the surgical process^5^. In the realm of interventional surgery, practitioners encompass a diverse background, including both surgeons and radiologists. Surgeons may possess limited expertise in the domain of radiology, while here, ChatGPT emerges as a valuable ally by providing guidance in the realm of image analysis. Conversely, radiologists may encounter a knowledge gap in interventions like RFA, MWA, and TACE, necessitating systematic training to enhance their proficiency, especially in clinical skills training. In this context, ChatGPT holds the potential to foster the dissemination and popularization of expertise across these intricate domains. In the field of healthcare, the integration of medical devices and artificial intelligence, such as combining with ChatGPT, is entirely feasible. Taking the example of RFA for liver cancer guided by ultrasound, many hospitals in China resort to traditional CT-guided RFA due to the limited ultrasound expertise of surgical practitioners. However, CT-guided RFA lacks real-time capability, leading to prolonged surgical times, increased patient discomfort, and potential radiation hazards. By synergizing ultrasound guidance with ChatGPT, the deficiencies arising from surgeons’ limited proficiency in utilizing ultrasound for interventions can be effectively addressed. This amalgamation allows surgeons to perform ultrasound-guided procedures more accurately, resulting in enhanced procedural efficiency and improved patient experience. ChatGPT can provide real-time guidance and support, assisting surgeons in making informed decisions to mitigate surgical duration and patient discomfort. This innovative integration holds significant implications for elevating surgical quality, expediting patient recovery, and reducing surgical risks.

This comprehensive approach marks a significant step forward in addressing the increasing complexities of oncological cases. As the interplay between ultrasound-guided interventions and artificial intelligence gains prominence, it reinforces the commitment to precision medicine. This dynamic synergy between medical expertise and technological advancements not only ensures better patient outcomes but also fosters an innovative ecosystem for continued development. In conclusion, the fusion of ultrasound guidance and artificial intelligence, exemplified by the integration of ChatGPT, represents an evolution in medical practice. This integration, driven by the shared goals of accurate diagnosis, personalized treatment, and improved patient experience, propels the field of interventional oncology toward a future of enhanced efficacy and patient-centric care. As we navigate this promising trajectory, it is crucial to maintain a balance between innovation and validation through rigorous clinical research, ensuring that these transformative approaches deliver on their potential to revolutionize oncological care (Fig. 1).

**Figure 1:**
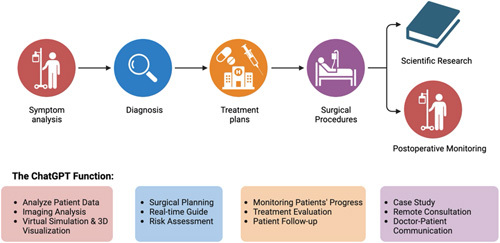
ChatGPT function in interventional surgery.

## Ethical approval

There is no need for ethical approval.

## Consent

There is no need for ethical approval.

## Sources of funding

None.

## Author contribution

These authors have contributed equally to this work.

## Conflicts of interest disclosure

There are no conflicts of interest for all authors.

## Research registration unique identifying number (UIN)

There is no need for UIN.

## Guarantor

Zhaorong Wu.

## Data availability statement

The data underlying this article will be shared by the corresponding author on reasonable request.

## Provenance and peer review

Not commissioned.
